# Elevated NIS Expression Correlates with Chemoresistance in Triple-Negative Breast Cancer: Potential Link to FOXA1 Activity

**DOI:** 10.3390/medsci13040250

**Published:** 2025-10-30

**Authors:** Grigory Demyashkin, Anastasia Guzik, Mikhail Parshenkov, Dmitriy Belokopytov, Vladimir Shchekin, Maxim Batov, Petr Shegai, Andrei Kaprin

**Affiliations:** 1 Department of Digital Oncomorphology, National Medical Research Centre of Radiology, 2nd Botkinsky Pass., 3, Moscow 125284, Russia; doctor.guzik@mail.ru (A.G.); misjakj@gmail.com (M.P.); belokopytov.sci@proton.me (D.B.); dr.shchekin@mail.ru (V.S.); md.batov@gmail.com (M.B.); dr.shegai@mail.ru (P.S.); kaprin@mail.ru (A.K.); 2 Laboratory of Histology and Immunohistochemistry, Institute of Translational Medicine and Biotechnology, I.M. Sechenov First Moscow State Medical University (Sechenov University), Trubetskaya st., 8/2, Moscow 119048, Russia; 3 Research and Educational Resource Center for Immunophenotyping, Digital Spatial Profiling and Ultrastructural Analysis Innovative Technologies, Peoples’ Friendship University of Russia (RUDN University), Miklukho-Maklaya st., 6, Moscow 117198, Russia; 4 Department of Urology and Operative Nephrology, Peoples’ Friendship University of Russia (RUDN University), Miklukho-Maklaya st., 6, Moscow 117198, Russia

**Keywords:** sodium iodide symporter (NIS), triple-negative breast cancer (TNBC), neoadjuvant chemotherapy, chemoresistance, FOXA1, Residual Cancer Burden (RCB), biomarker

## Abstract

Background: Sodium/iodide symporter (NIS) is a membrane protein involved in iodide transport into cells, making it a key component of thyroid physiology and radioiodine therapy for thyroid cancer. Although NIS is expressed in many extrathyroidal tissues, including breast tumors, its functional role and prognostic significance in these contexts remain a subject of active investigation. Understanding the mechanisms regulating NIS, its influence on cellular processes such as migration and metastasis, and its connection with transcription factors like FOXA1 could contribute to the development of new therapeutic strategies for breast cancer treatment. This study aims to investigate the correlation between sodium/iodide symporter (NIS) expression and response to neoadjuvant chemotherapy in patients with triple-negative breast cancer (TNBC). Methods: The current retrospective study included 161 TNBC patients who received neoadjuvant chemotherapy followed by mastectomy. NIS expression was assessed via immunohistochemistry, graded semi-quantitatively from 0 to 3+. The Residual Cancer Burden (RCB) scale was used to evaluate the response to chemotherapy. Statistical analysis included Lilliefors tests and Kendall’s tau correlation coefficient. Publicly available Cancer Genome Atlas datasets were analyzed to assess the relationship between NIS and FOXA1 expression. Results: NIS immunopositivity was observed in 69.5% of TNBC samples compared to 63.3% GATA-3-positive and 31.0% of Mammaglobin-positive samples. While no significant correlation was found between NIS expression and age, TNM stage, or Ki-67, a statistically significant moderate positive correlation (τ = 0.481, *p* < 0.01) was identified between NIS expression and RCB index, indicating that higher NIS expression was associated with a poorer response to neoadjuvant chemotherapy. TCGA data analysis revealed a statistically significant increase in NIS mRNA expression in FOXA1-mutated TNBC samples compared to FOXA1-wild-type samples (*p* < 0.05). Younger patients exhibited higher Ki-67 levels (τ = −0.416, *p* < 0.05). Conclusions: Higher NIS expression correlates with chemoresistance to neoadjuvant chemotherapy in TNBC patients. This phenomenon may be linked to FOXA1 activity, suggesting that NIS may represent a potential biomarker for chemoresistance in TNBC. The inverse correlation between patient age and Ki-67 levels may be associated with a different mutational landscape in younger patients.

## 1. Introduction

Sodium/iodide symporter (NIS) is a membrane glycoprotein consisting of 643 amino acids, the sequence of which is encoded by the *SLC5A5* gene located on the short arm of chromosome 19—19p12-13.2 [1]. The main function of this protein is the co-transport of one molecule of iodide with two molecules of sodium, driven by the membrane potential difference created by the Na+/K+-ATPase [2]. The study of NIS properties was primarily related to research in the field of thyroid physiology and metabolism, as well as radiopharmaceutical therapy for thyroid malignancies [3]. Thus, one of the most successful and actively used methods to date for treating thyroid carcinomas is radioiodine therapy (I131), which allows for visualization using scintigraphy and targeted destruction of radioiodine-avid tumor cells [4]. Visualization of NIS-expressing cells using a radiopharmaceutical and scintigraphy forms the basis for currently actively developed virotherapy and mesenchymal stem cell therapy approaches, where transfection with the NIS gene allows for tracking the spread of the oncolytic viral vector throughout the body, as well as assessing the degree of virus propagation in the tumor [5]. In addition, NIS allows for radioviropharmacotherapy, enhancing the oncolytic effect of the virus by administering therapeutic radioiodine [6]. For example, the application of the MV-NIS (NIS-modified Measles Virus) strain has been extremely successful, demonstrating promising results in the treatment of patients with chemotherapy-resistant ovarian tumors and pediatric medulloblastoma [7,8].

The use of NIS-directed oncolytic virotherapy methods is primarily because most tumors either do not express NIS themselves or express it in a therapeutically insignificant amount, or because NIS expression is non-functional because the symporter is not incorporated into the tumor cell plasma membrane and, therefore, is unable to capture radioiodine [9,10]. The latter variant is characteristic of breast carcinomas [11]. In 2000, Tazebay et al. discovered that most (approximately 80%) breast carcinomas have pronounced cytoplasmic NIS expression, which initiated many studies in this area, the interim result of which can be considered the work of Peyrottes et al., 2009, which presents arguments in favor of the non-specific nature of staining with NIS antibodies and a significantly lower actual level of expression of this protein [12,13]. Later studies in this area, however, indicate that NIS is a dimer consisting of a main N-terminal part and an intracellular C-terminal domain, and most of the antibodies used by researchers target the C-terminal domain, the detection of which, using Western blot, is difficult due to its low molecular weight, which, in our opinion, may distort the reality of NIS expression [14]. Despite this, there is currently no convincing data indicating the ability of breast carcinomas to capture radioiodine in vivo in a therapeutic amount, which has prompted researchers to study the role of NIS in tumor biology, the mechanisms of its expression, processing, transport, and glycosylation [15].

One particularly interesting study is the article by Lacoste et al., which convincingly demonstrates the pro-oncogenic role of intracytoplasmic NIS promoting tumor cell migration and metastasis by interacting with the guanine nucleotide exchange factor of Ras Homolog Family Member A Guanosine Triphosphatase (RhoA GTPase) involved in cell adhesion. In this case, NIS acts more as a regulator, preventing Leukemia-Associated Rho Guanine Nucleotide Exchange Factor (LARG) from binding to RhoA GTPase [16,17]. Furthermore, it is known that NIS expression is regulated by, amongst other things, the activity of p53, which suppresses NIS expression. Thus, TP53-mutant breast carcinomas express NIS more actively [18]. Similar data were obtained by Rathod et al. for the transcription factor FOXA1, the activity of which directly affects NIS expression [19]. In addition, there is evidence indicating the role of the *FOXA1* gene in the development of chemoresistance and the existence of a FOXA1-positive chemotherapy refractory triple-negative breast cancer (TNBC) subtype [20,21,22]. The data described above reliably suggest a significant, if not a key, role for NIS in tumor biology; therefore, the aim of our study is to investigate the features of NIS expression in TNBC and the correlation of the degree of expression with clinically important prognostic parameters, allowing for the potential use of NIS as a useful biomarker.

This study aims to investigate the correlation between sodium/iodide symporter expression and response to neoadjuvant chemotherapy in patients with triple-negative breast cancer.

## 2. Material and Methods

### 2.1. Patients

In this retrospective study, the medical records from 2020 to 2023 of patients treated at the Department of Oncology and Reconstructive-Plastic Surgery of the Mammary Gland and Skin of the P.A. Herzen Institution were analyzed. Among 2719 cases diagnosed in our center, 161 patients were enrolled according to the criteria outlined in Table 1.

### 2.2. Clinical Data

All patients included in the study cohort, based on the decision of the oncology board, were selected for a treatment strategy involving neoadjuvant chemotherapy according to the listed protocols, followed by mastectomy including doxorubicin (A), cyclophosphamide (C), docetaxel (T), paclitaxel (P), carboplatin (Carbo), and a dose-dense regimen (dd):

4AC+4T/12P (Doxorubicin 60 mg/m^2^ IV on day 1 + cyclophosphamide 600 mg/m^2^ IV on day 1 every 3 weeks for 4 cycles + docetaxel 75 mg/m^2^ IV on day 1 every 3 weeks for 4 cycles OR paclitaxel 80 mg/m^2^ IV weekly for 12 weeks).

4ddAC+12P (Dose-dense: Doxorubicin 60 mg/m^2^ IV on day 1 + Cyclophosphamide 600 mg/m^2^ IV on day 1, cycled every 14 days for four cycles, followed by paclitaxel 80 mg/m^2^ IV weekly for 12 weeks).

4ddAC+4ddP (Dose-dense: Doxorubicin 60 mg/m^2^ IV on day 1 + Cyclophosphamide 600 mg/m^2^ IV on day 1, cycled every 14 days for four cycles, followed by Paclitaxel 175 mg/m2 IV day 1 cycled every 14 days for four cycles).

4ddAC+P+Carbo (Dose-dense: Doxorubicin 60 mg/m^2^ IV on day 1 + Cyclophosphamide 600 mg/m^2^ IV on day 1, cycled every 14 days for four cycles, followed by paclitaxel 80 mg/m^2^ IV weekly for 12 weeks with Carboplatin AUC 6 IV day 1).

A decision on the number of chemotherapy cycles and the initiation of surgery was also made based on the conclusion of the oncology board.

### 2.3. Real-Time Polymerase Chain Reaction BRCA1/2 and CHEK2 Mutation Analysis

Venous blood samples were obtained from all patients participating in the study and subsequently analyzed for *BRCA1/2* gene mutations using real-time polymerase chain reaction (DTPrime4; DNA-technology). Investigated mutations of *BRCA1* gene: 5382insC (insertion of C at position 5382); 4153delA (deletion of A at position 4153); 300T/G (substitution from T to G at position 300); 3819delGTAAA (deletion of GTAAA at position 3819); 2080delA (deletion of A at position 2080); 185delAG (deletion of AG at position 185); and 3875delGTCT (deletion of GTCT at position 3875). *BRCA2* gene: 6174delT (deletion of T at position 6174). Germline mutation analysis of the *CHEK2* gene associated with breast and ovarian cancer was performed. Investigated mutations: 1100delC (deletion of C at position 1100); IVS2+1G>A (substitution from G to A at position +1 of Intron 2 Splicing Site); and 470T>C (substitution from T to C at position 470).

### 2.4. Morphological Block

Breast tissue fragments were fixed in buffered formalin solution, processed automatically, embedded in paraffin blocks, and sectioned into serial slices with a thickness of 2 μm. The sections were then deparaffinized, dehydrated, and stained with hematoxylin and eosin. The evaluation of the specimens was conducted according to standard histological criteria: The Nottingham Histologic Score was used to assess the grade of malignancy (Table 2).

Surrogate molecular-genetic phenotype was evaluated using WHO and College of American Pathologist recommendations using Allred system and St. Gallen’s consensus criteria. A triple-negative phenotype was considered to be 2 or fewer points using Allred for Estrogen Receptor (ER) and Progesterone Receptor (PR) as well as the absence of Human Epidermal Growth Factor Receptor 2 (HER2/neu) expression (Table 3, Table 4 and Table 5). When assessing proliferative activity, at least 1000 tumor cells were analyzed, and counting was performed on all tumor material, taking into account “hot spots” [21]. Immunohistochemical staining was performed, and the following primary antibodies were used: monoclonal antibodies against Sodium/Iodide Symporter (NIS/SL5A5; Affinity Biosciences Cat# DF2242, RRID: AB_2839473); Mammaglobin (clone 31A5 CellMarque), GATA-3 (clone L50-823 CellMarque), anti-Estrogen Receptor (ER clone SP1 Ventana), anti-Progesteron Receptor (PgR, clone 1E2 Ventana), anti-HER2/NEU (clone 4B5 Ventana), and anti-Ki-67 (clone 30-9 Ventana). For secondary antibody detection, a universal two-component HiDef Detection™ HRP Polymer system (Cell Marque, Rocklin, CA, USA) was used, including anti-IgG mouse/rabbit antibodies, horseradish peroxidase (HRP), and DAB substrate. Cell nuclei were counterstained using Mayer’s hematoxylin. Fragments of thyroid gland with Graves’ disease (staining of follicular cells) [23] were used as an external control for immunohistochemical examination with antibodies to NIS, skin fragments (staining of sweat glands) [24] were used for reaction with antibodies to Mammaglobin, and cervical fragments (staining of basal and parabasal cells of the exocervical epithelium) [25] were used for reaction with antibodies to GATA-3.

Based on the results of the immunohistochemical staining, the surrogate molecular-genetic subtype was determined according to the expression profile of hormone receptors (Table 5).

NIS staining intensity was graded using modified Gainor’s semi-quantitative method from 0 to 3+, according to our grading system as follows:

0 points—Staining absent.

1 point—Pale cytoplasmic staining, distinguishing positive cells from the background, fine granules present.

2 points—Distinct cytoplasmic staining, with a coarse granular staining pattern.

3 points—Prominent membrane-cytoplasmic staining, with coarse granular staining; individual granules are often not discernible [26].

**Figure 1:**
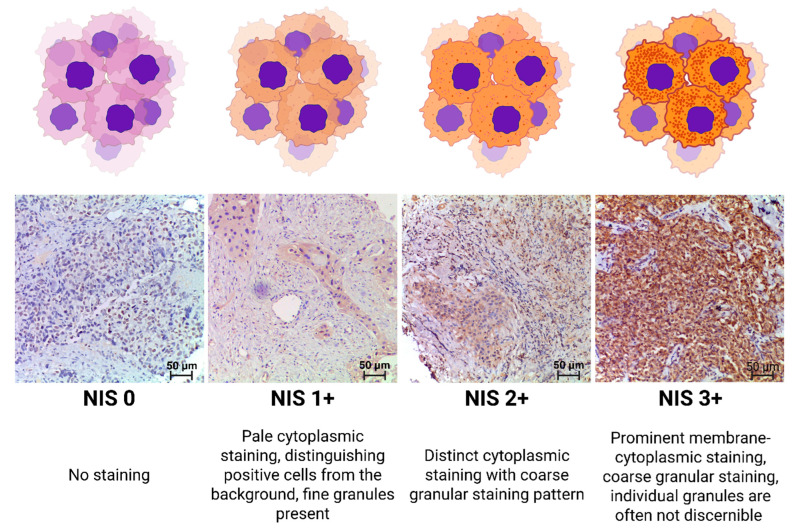
NIS expression-level criteria.

A visual representation of the applied scale is shown in Figure 1. Microscopic analyses were conducted utilizing a video microscopy system comprising a Leica DM3000 microscope (Leica Microsystems, Wetzlar, Germany), a Leica ICC50 HD camera (Leica Camera, Wetzlar, Germany), and a Platrun LG computer (LG Electronics Inc., Seoul, Republic of Korea).

### 2.5. Assessment of Residual Cancer Burden (RCB)

Regarding the surgical material obtained during mastectomy by the resection of regional lymph nodes, the response to chemotherapy treatment was assessed using the MD Anderson Residual Cancer Burden (RCB) scale, dividing patients into 4 groups depending on the severity of the response: RCB-0 (RCB score 0, equivalent to complete response), RCB-1 (RCB score ≥ 0–1.36), RCB-2 (RCB score 1.37–3.28), and RCB-3 (RCB score > 3.28). The calculation was performed using the RCB calculator available online (www.mdanderson.org/breastcancer_RCB, accessed on 20 September 2025) [27].

### 2.6. Statistical Analysis

Statistical analysis of the samples was performed using Statistica 13.5.0.17 software (TIBCO Software Inc., San Ramon, CA, USA). The description of quantitative data included the determination of the median and interquartile range (IQR; 25–75 percentile) or mean and standard deviation (SD), depending on the normality of the distribution (Lilliefors test: *p* > 0.20); *p*-value ≤ 0.05 was considered statistically significant. The degree of correlation between variables was assessed using Kendall’s tau rank correlation coefficient. Kendall’s tau (τ) correlation coefficients were interpreted based on standard guidelines: τ < 0.2 as negligible/weak, 0.2–0.4 as weak/low, 0.4–0.6 as moderate, 0.6–0.8 as strong, and >0.8 as very strong [28].

### 2.7. TCGA and METABRIC Data Analysis of NIS and FOXA1 Expression

The Cancer Genome Atlas and METABRIC data were curated from cBioPortal (http://www.cbioportal.org/index.do, accessed on 20 September 2025). Statistical analysis was performed using proprietary cBioPortal tests [29,30]. Differences were considered statistically significant at *p* ≤ 0.05. The q-value (FDR-adjusted *p*-value) was reported as an additional measure to account for multiple comparisons, with q ≤ 0.05 indicating robust significance after correction. In cases where *p* < 0.05 but q > 0.05, results were interpreted as suggestive trends, warranting further validation, rather than definitive findings.

## 3. Results

### 3.1. Patients

**Figure 2:**
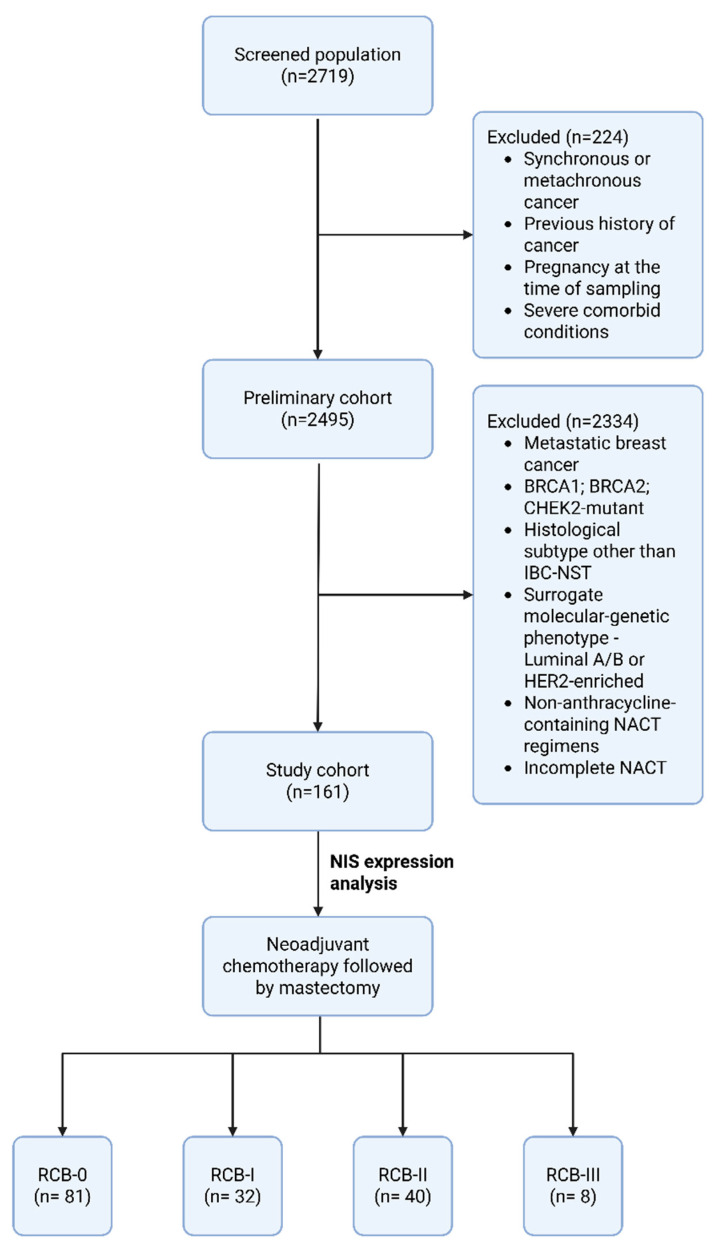
Study flowchart. Abbreviations: IBC-NST—Invasive Breast Carcinoma of no special type; NACT—neoadjuvant chemotherapy; RCB—residual cancer burden.

The final cohort consisted of 161 patients (Figure 2). Patients’ medical records were analyzed. The main clinical data for the patient cohort are presented in Table 6. The average age of patients in the obtained sample was 53.6 years (SD: ±11.97). Among them, 75.7% of patients (n = 122) were older and 24.3% were younger than 45 years (n = 39).

### 3.2. Clinical Data

All patients included in the study cohort, based on the decision of the oncology board, underwent neoadjuvant anthracycline-based chemotherapy according to the following protocols:

4AC+4T/12P (Doxorubicin 60 mg/m^2^ IV on day 1 + cyclophosphamide 600 mg/m^2^ IV on day 1 every 3 weeks for 4 cycles + docetaxel 75 mg/m^2^ IV on day 1 every 3 weeks for 4 cycles OR paclitaxel 80 mg/m^2^ IV weekly for 12 weeks).

4ddAC+12P (Dose-dense: Doxorubicin 60 mg/m^2^ IV on day 1 + Cyclophosphamide 600 mg/m^2^ IV on day 1, cycled every 14 days for four cycles, followed by paclitaxel 80 mg/m^2^ IV weekly for 12 weeks).

4ddAC+4ddP (Dose-dense: Doxorubicin 60 mg/m^2^ IV on day 1 + Cyclophosphamide 600 mg/m^2^ IV on day 1, cycled every 14 days for four cycles, followed by Paclitaxel 175 mg/m2 IV day 1 cycled every 14 days for four cycles).

4ddAC+P+Carbo (Dose-dense: Doxorubicin 60 mg/m^2^ IV on day 1 + Cyclophosphamide 600 mg/m^2^ IV on day 1, cycled every 14 days for four cycles, followed by paclitaxel 80 mg/m^2^ IV weekly for 12 weeks with Carboplatin AUC 6 IV day 1).

All therapies included subsequent mastectomy. The final number of courses of neoadjuvant chemotherapy corresponded to the planned number, with assessments of effectiveness based on the results of computed tomography. Based on the results obtained, patients were then referred for surgery in the scope of total mastectomy with removal of regional lymph nodes of the axillary adipose tissue. All patients were alive at the start of the study.

### 3.3. Real-Time Polymerase Chain Reaction BRCA1/2 and CHEK2 Mutation Analysis

Genetic testing of peripheral blood samples using real-time polymerase chain reaction (DTPrime4; DNA-technology) in patients (n = 161) revealed no mutations in the *BRCA1*, *BRCA2*, or *CHEK2* genes.

### 3.4. Morphologic Examination

**Figure 3:**
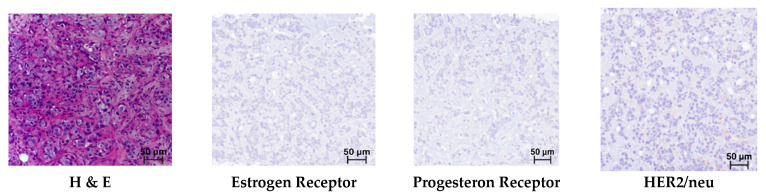

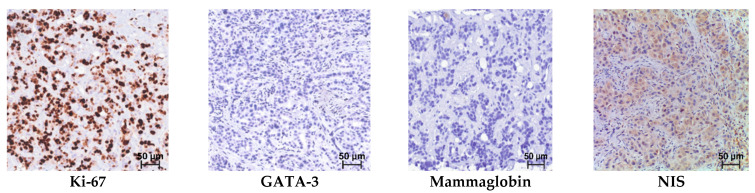
Triple-negative breast cancer. Hematoxylin and eosin (H & E) staining and immunohistochemical reactions with antibodies; mag. ×250.

According to the results of the histological examination, all core biopsy samples of mammary glands (n = 161) revealed a typical morphological picture of invasive carcinoma of non-specific type (IBC-NST), which is Grade 3 according to the Nottingham Grading system. Microfragments of the mammary gland with pathologically altered tissue (tissue and cellular atypia of the parenchyma): a cluster of pleomorphic atypical cells (>300) forming solid structures. The cytoplasm of these cells is in the form of a thin rim; their nuclei with signs of polymorphism are located eccentrically, and they are rounded in shape, with an “eroded” karyolemma. Stromal component with a weak desmoplastic reaction (Figure 3).

In the immunohistochemical study of breast biopsy material from patients (n = 161), no reaction was detected with antibodies to the Estrogen Receptor (Allred = 0), Progesteron Receptor (Allred = 0), or HER2/neu (–/0), which corresponds to triple-negative cancer (Figure 3).

In the immunohistochemical study observing antibodies to NIS, Mammaglobin, and GATA-3, positive reactions were observed in almost all samples; however, there were cases that were positive only in the reaction with NIS (Figure 3). The distribution of immunopositive reactions in atypical cells of triple-negative breast cancer among patients, depending on the marker, was as follows: NIS-positive reactions were found in 112 women (69.5%); Mammaglobin in 50 (31.0%); and GATA-3 in 102 (63.3%). Some patients showed staining for two or more antibodies, which accounted for 83.7% of cases. Immunopositive reaction with only one marker was observed in ten women: of these, four were NIS-positive only; four were GATA-3 only; and two were Mammaglobin only. Only 27 (16.7%) women showed no staining with antibodies to NIS, Mammaglobin, or GATA-3.

**Figure 4:**
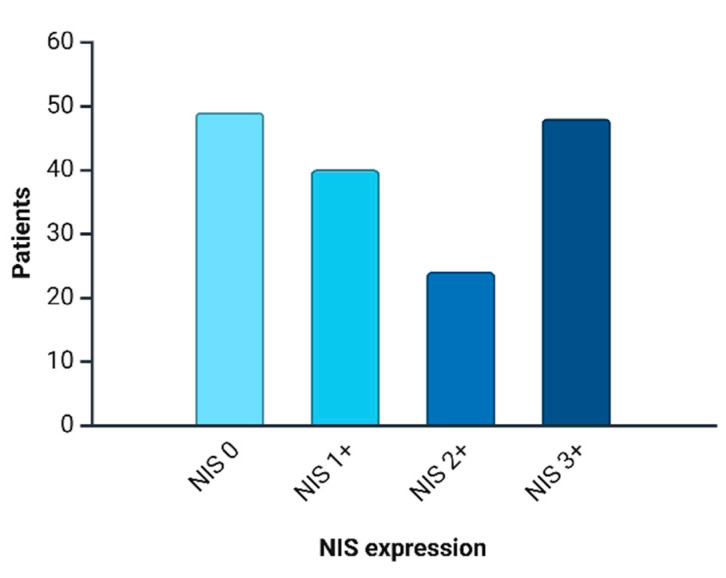
NIS expression distribution in current cohort.

NIS immunopositive reactions were found in 112 (69.5%) patients, and the intensity distribution was as follows: NIS 1+ was observed in 40 (35.7%) cases; NIS 2+ in 24 (21.4%) cases; and NIS 3+ in 48 (42.8%) cases. Triple-negative breast cancer was NIS-negative in 49 (30.4%) women. The expression level distribution is depicted in Figure 4.

**Figure 5:**
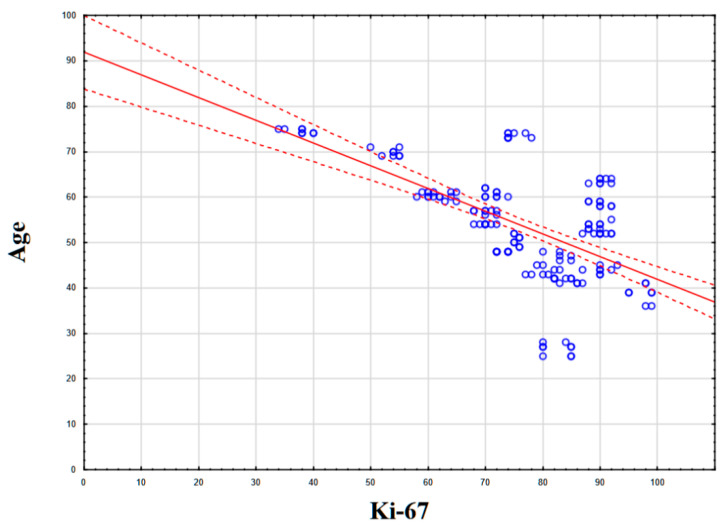
Statistical correlation between Ki-67 levels (%) and patient age.

Statistical analysis of the correlation between NIS expression and patient age, the number of affected lymph nodes, the level of mitotic activity, and the level of Ki-67 revealed no statistically significant correlation (*p* > 0.05). However, a statistically significant correlation was found between patient age and the level of proliferative activity Ki-67 (%): the Kendall tau coefficient was −0.416 (*p* < 0.05), which corresponds to a weak–moderate negative association (Figure 5).

**Figure 6:**
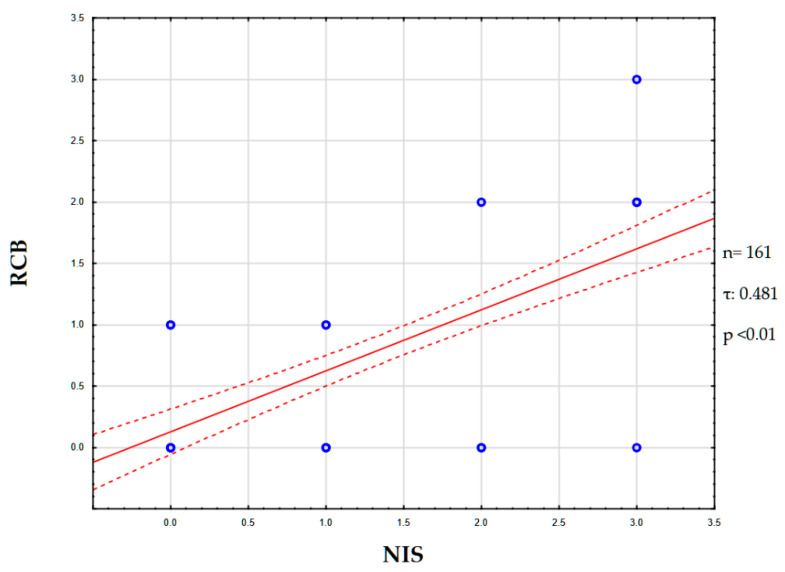
NIS and RCB correlation graph. Abbreviations: NIS—sodium/iodide symporter; RCB—residual cancer burden.

A correlation was found between the degree of NIS expression and the Residual Cancer Burden (RCB) index. Thus, the Kendall tau coefficient was 0.481 (*p* < 0.01), which corresponds to a moderate positive association. Thus, higher NIS expression corresponded to an increased tumor resistance to neoadjuvant chemotherapy (Figure 6).

### 3.5. TCGA Data Analysis of NIS and FOXA1 Expression

We hypothesized that the correlation between NIS expression and the degree of response to chemotherapy could be explained by the presence of driver or amplifying mutations in the *FOXA1* gene, the activity of which is characterized by the formation of a chemoresistant tumor phenotype.

To test our hypothesis, we turned to open data from the cBioportal resource, namely the two largest studies with mRNA expression data, BRCA TCGA Firehouse Legacy and BRCA TCGA Cell.

**Figure 7:**
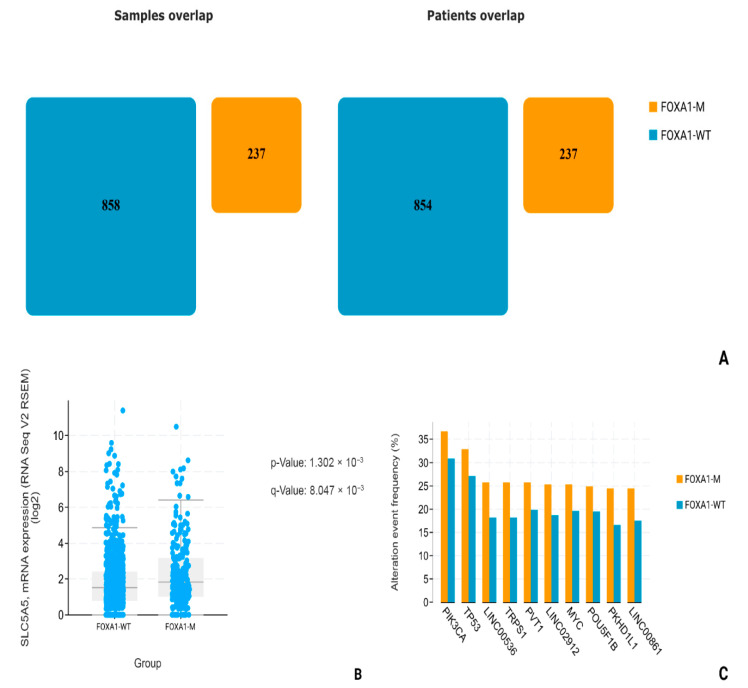
Statistical analysis of TCGA, Firehouse dataset. (**A**) FOXA1-M and FOXA1-WT number of patients. (**B**) SLC5A5 mRNA levels comparison. (**C**) Most common associated mutations in FOXA1-M and FOXA1-WT groups.

Using data from patients (n = 1091) obtained during the Breast Invasive Carcinoma (TCGA, Firehose Legacy) study, we conducted a comparative analysis of NIS (SLC5A5) mRNA expression in two subgroups, the FOXA-1-Wild-type (FOXA1-WT; n = 854) and FOXA1-mutated group, including driver mutations, gain mutations, and amplifications (FOXA1-M; n = 237). All overlapping samples were excluded from the study (Figure 7A). During the analysis, we found a statistically significant increase in NIS mRNA expression in the FOXA1-M subgroup compared to FOXA1-WT (*p*-value: 1.302 × 10^−3^; q-value: 8.047 × 10^−3^) (Figure 7B). In addition, we assessed the group frequency of TP53 co-mutation to exclude the influence of mutations in this gene on our results. No statistically significant differences in the frequency of co-mutations in TP53 were found (*p*-value: 0.133; q-value: 0.821) (Figure 7C).

**Figure 8:**
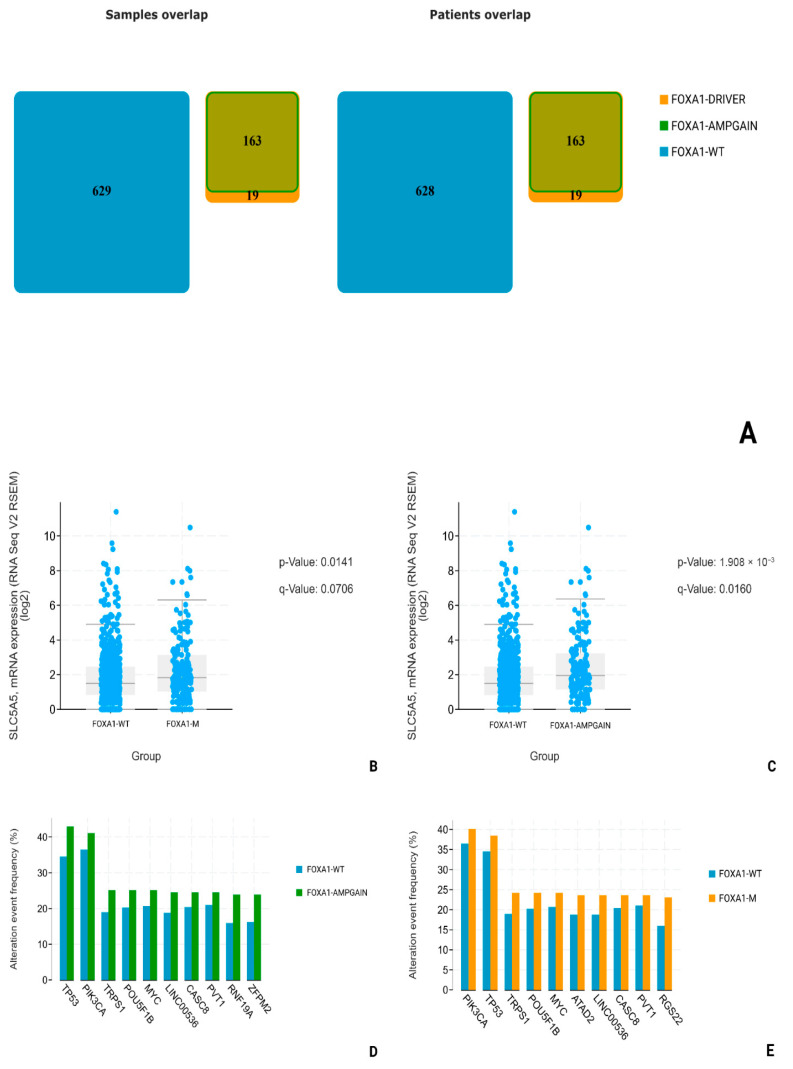
Statistical analysis of TCGA—cell dataset. (**A**) FOXA1-M (FOXA1-DRIVER + FOXA1-AMPGAIN) and FOXA1-WT number of patients. (**B**) SLC5A5 mRNA levels comparison in FOXA1-WT and FOXA1-M groups. (**C**) SLC5A5 mRNA levels comparison between FOXA1-WT and FOXA1-AMPGAIN groups. (**D**) Most common associated mutations in FOXA1-WT and FOXA1-AMPGAIN groups. (**E**) Most common associated mutations in FOXA1-WT and FOXA1-M groups.

Using data from patients (n = 820) obtained during the Invasive Breast Carcinoma (TCGA. Cell 2015) study, we conducted a comparative analysis of NIS (SLC5A5) mRNA expression in two groups: FOXA1-WT (n = 627) and FOXA1-M (n = 182). All overlapping samples were excluded from the study (Figure 8A). During the analysis, we found a suggestive increase in NIS mRNA expression in the FOXA1-M group compared to the *FOXA1*-WT group (*p*-Value: 0.0141; q-Value: 0.0706) and subsequent highlighting of a separate subgroup only with amplification and gain-mutations FOXA1-AMPGAIN (n = 163); the degree of statistical significance of differences in NIS mRNA expression compared to the FOXA1-WT group was higher (*p*-Value: 1.902 × 10^−3^; q-Value: 0.0160) (Figure 8B,C). In addition, we assessed the frequency of the most associated mutations in all three subgroups, and no statistically significant differences in the frequency of TP53 mutations were found, neither between the FOXA1-WT and FOXA1-M groups (*p*-value: 0.379; q-value: 1.00), nor between the FOXA1-WT and FOXA1-AMPGAIN groups (*p*-value: 0.0277; q-value: 0.521) (Figure 8D,E).

To further assess the specificity of NIS-FOXA1 associations in TNBC, we performed a subgroup analysis restricted to triple-negative cases (defined as ER-negative, PR-negative, and HER2-negative) within the TCGA datasets. In the Breast Invasive Carcinoma (TCGA, Firehose Legacy) dataset, the TNBC subgroup comprised n = 69 samples, among which only 1 case exhibited a FOXA1 mutation (with no available mRNA expression data for NIS/SLC5A5), while the remaining samples were FOXA1 wild-type. Similarly, in the Invasive Breast Carcinoma (TCGA, Cell 2015) dataset, the TNBC subgroup included n = 51 samples, with FOXA1 mutations identified in only 2 cases. No statistically significant correlation was observed between FOXA1 mRNA levels and NIS/SLC5A5 mRNA expression in these subgroups (*p* > 0.05). Notably, TP53 mutations were prevalent in >80% of these TNBC samples, consistent with known genomic profiles of this subtype [18], which may confound the detection of FOXA1-NIS interactions.

## 4. Discussion

In this study, we analyzed the degree of NIS expression in biopsy samples from patients (n = 161) with triple-negative breast cancer. Of these, 69.5% (n = 112) were NIS-positive, Mammaglobin-positive—31.0% (n = 50), and GATA-3-positive—63.3% (n = 102). NIS can be considered one of the sensitive markers of breast cancer, as its expression persists even in poorly differentiated TNBC. Taken together with data from other authors, the specificity of NIS is comparable to that of Mammaglobin. However, a significant advantage of NIS is the absence of its expression in healthy breast tissue, unlike other specific markers of breast cancer [31,32]. This fact is of significant diagnostic importance, especially in the verification of cancer of unknown primary. The degree of NIS expression varied significantly; we can note the predominance of NIS 3+ expression type—48 (42.8%), the second most frequent was the NIS 1+ phenotype—40 (35.7%), and the NIS 2+ phenotype was even rarer—24 (21.4%). During the analysis of the degree of correlation between NIS expression and clinical parameters, we did not find a statistical relationship with TNM stages, age, mitotic or proliferative activity of the tumor (Ki-67), etc. Statistically significant was the correlation with the level of residual cancer burden (RCB), assessed after neoadjuvant chemotherapy and mastectomy [33]. The degree of correlation estimated using the Kendall tau coefficient was 0.481, which corresponds to a moderate positive association, i.e., higher NIS expression is characteristic of chemoresistant variants of TNBC. It is not currently possible to assess differences in mortality and survival rates of patients, as all patients included in our study were alive at the last follow-up (36 months). Chekun et al. gained similar results in their work, finding a correlation between NIS expression and mortality in patients with Luminal B and Basal subtypes [34].

The explanation for the differences in the degree of NIS expression and the response to chemotherapy lies in the molecular genetic characteristics of hormone-negative breast carcinomas. As is known, TNBC is an umbrella term that unites extremely heterogeneous groups of tumors, differing both in the degree of mutational burden and in key driver mutations. In the analysis of the literature, we found the work of Rathod et al., in which it was experimentally demonstrated on cell cultures that FOXA1 is one of the key regulators of NIS expression [19]. In order to analyze whether a similar relationship between FOXA1 activity and NIS expression is found in patients in clinical practice, we turned to open datasets on the cBioPortal resource. Choosing the two largest datasets with information on mRNA expression obtained by sequencing (TCGA, Cell and TCGA, Firehouse legacy), we compared the levels of NIS (SLC5A5) mRNA in groups with FOXA1-wild-type (without mutations) and FOXA1-MUT (driver mutations, increased number of gene copies). In both datasets, we found a statistically significant increase in NIS mRNA levels in the group with mutant FOXA1. In addition, for reliability, it was necessary to exclude the influence of mutations in TP53, since it is known that non-mutant forms of p53 suppress NIS expression. We analyzed the frequency of TP53 mutations in the FOXA1-WT and FOXA1-MUT groups and found no statistically significant differences, thus excluding the possible influence of differences in TP53 expression on our result. Although TCGA analysis was performed across all subtypes due to limited TNBC sample size, subgroup analysis revealed no NIS-FOXA1 correlation in TNBC, likely confounded by TP53 mutations. Future studies in larger TNBC cohorts are warranted.

**Figure 9:**
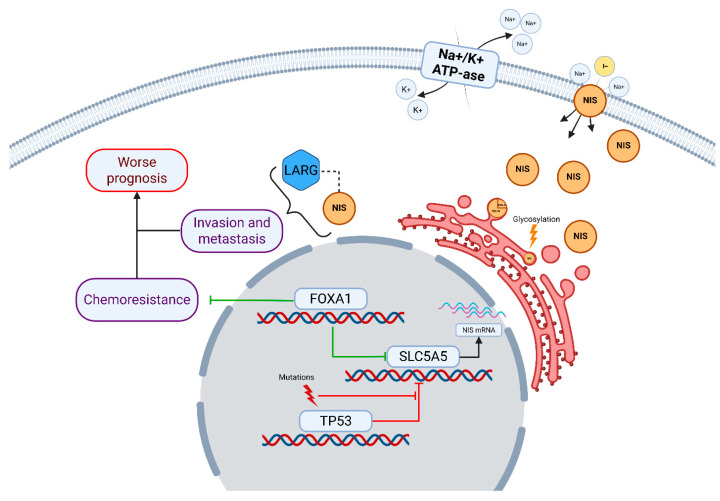
Implication of NIS in breast cancer pathogenesis. NIS/SLC5A5 is shown as a membrane glycoprotein facilitating Na+/I− co-transport, driven by the Na+/K+-ATPase gradient. Intracytoplasmic NIS interacts with LARG, promoting RhoA GTPase activation, which enhances cell invasion and metastasis, contributing to worse prognosis [16,17]. NIS expression is regulated by transcription factors: FOXA1 upregulates NIS transcription, potentially leading to chemoresistance in FOXA1-mutated or amplified tumors [19,20,21], while TP53 mutations suppress NIS repression, increasing its levels [18]. Additional post-translational modifications, such as glycosylation, affect NIS localization and function [10,14]. Mutations in TP53 and FOXA1 are depicted as drivers of dysregulated NIS expression, linking to chemoresistance pathways.

The exact way that FOXA1 influences tumor chemoresistance and prognosis is still under debate. In luminal, hormone-positive subtypes, FOXA1 expression is linked to better response to hormonal therapy. It also correlates with lower tumor malignancy and smaller size [35]. These positive effects may result from FOXA1’s modulation of estrogen receptors. In luminal B or triple-negative subtypes, tumor cells lack sufficient estrogen receptors. Here, other FOXA1 functions become important. As a transcription factor, FOXA1 can maintain a “luminal” tumor phenotype [35,36]. The luminal A subtype resists many cytostatic and cytotoxic drugs, such as paclitaxel and doxorubicin. According to the St. Gallen Consensus, chemotherapy is inappropriate for luminal A breast cancer [37]. In TNBC without estrogen receptors but with preserved FOXA1, cells may gain some luminal-like features. This could make them more resistant to chemotherapy [36]. A schematic implication of NIS in breast cancer pathogenesis is summarized in Figure 9.

**Figure 10:**
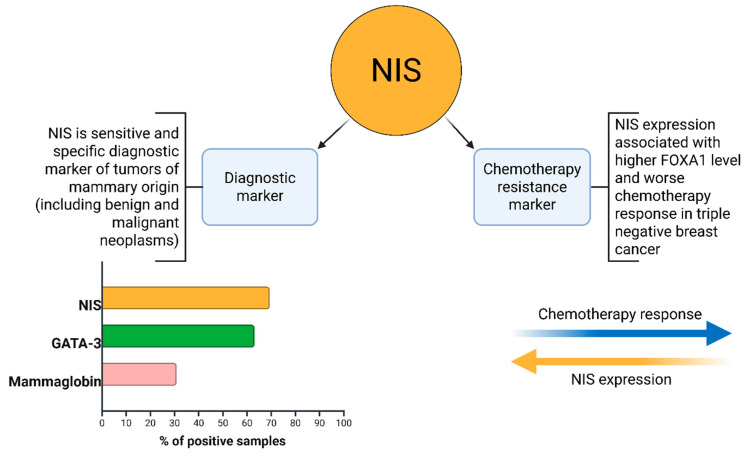
NIS clinicopathological implications.

NIS expression, in this case, is more of an indirect sign, reflecting the activity status of FOXA1, and, according to the work of Kelkar, TP53 [18]. Thus, we suggest that NIS can be used as an additional biomarker capable of indicating the expected response to chemotherapy in TNBC. Perhaps further research in the field of adapting radioiodine therapy to the treatment of malignant breast tumors will be able to use increased NIS expression for the treatment of NIS-expressing chemoresistant variants of TNBC. The main results of our study are summarized in Figure 10.

We consider it important to separately explain the reason for excluding all carriers of BRCA1, BRCA2, and CHEK2 mutations from our study. According to data from the TNT Trial, BRCA1/2 mutation carriers exhibit a better response to platinum-based drugs compared to taxanes: 66% objective response rate versus 33%. Therefore, their exclusion served to increase sample homogeneity and to mitigate potential biases in the interpretation of the response to chemotherapy; however, this also limits the extrapolation of our findings to this cohort of patients [38].

**Figure 11:**
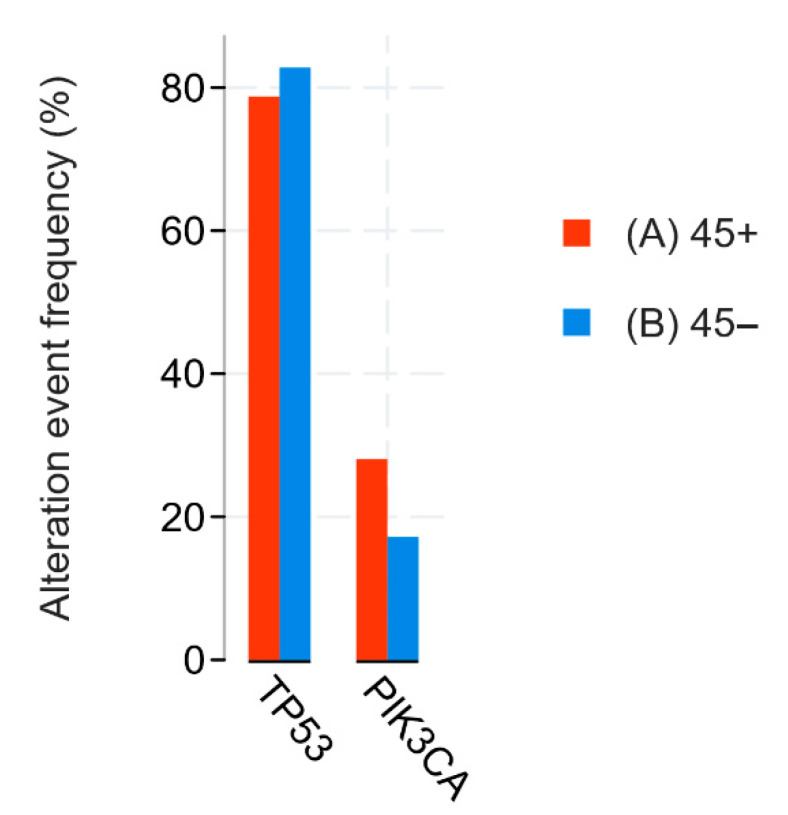
Statistical analysis of METABRIC data. TP53 and PIK3CA mutations comparison between age < 45 (blue) and age ≥ 45 (red) groups.

Revealing a correlation between patient age and tumor proliferative activity was not the aim of our study, but we found a statistically significant negative association between these two variables: the Kendall tau coefficient was −0.4166. Thus, higher proliferative activity of triple-negative breast cancer was characteristic of younger patients. The literature data on this issue are controversial. There are articles that indicate the presence of a statistically significant correlation [39,40,41], as well as articles presenting opposite conclusions [42,43]. Since our sample consisted only of patients with triple-negative breast cancer, we assume that the correlation obtained can be explained from the point of view of the mutational profile characteristics of breast cancer at a young age. Again, turning to the data cBioPortal Breast Cancer (METABRIC, Nature 2012 & Nat Commun 2016) dataset, in the TNBC cohort (n = 210), we found a higher frequency of TP53 mutations in the group of TNBC patients younger than 45 years (n = 64), 82.81%, versus 78,7% in women older than 45 years (n = 146) (Figure 11) [29,30]. It is reliably known that ***TP53*** mutations are associated with increased proliferative activity in tumors. Thus, according to the scientific literature, when comparing p53-mutant and p53-wt breast carcinomas, the Ki-67 index was higher in p53-mutant tumors by an average of 16% (51.77 ± 24.53 versus 35.81 ± 19.54) [44]. We hypothesize that the higher frequency of mutations in the ***TP53*** gene in young women is one of the key factors determining both the higher proliferative activity of the tumor and the greater frequency of occurrence of triple-negative breast cancer. From a clinical point of view, higher proliferative activity is a factor that negatively affects the patient’s prognosis and recurrence-free survival.

## 5. Conclusions

In conclusion, our study demonstrates that higher NIS expression correlates with increased chemoresistance to neoadjuvant chemotherapy in TNBC patients, as evidenced by a moderate positive correlation with the RCB index (τ = 0.481, *p* < 0.01). NIS immunopositivity was observed in 69.5% of samples, surpassing GATA-3 (63.3%) and Mammaglobin (31.0%) positivity. Additionally, a negative correlation between patient age and Ki-67 levels (τ = −0.416, *p* < 0.05) highlights age-related differences in tumor proliferative activity. These findings position NIS as a potential biomarker for chemoresistance in TNBC, warranting further validation in larger cohorts.

## Figures and Tables

**Table 1:** Inclusion and exclusion criteria.

Inclusion Criteria	Exclusion Criteria
Female gender Age ≥ 18 years Newly diagnosed breast cancer (≥T1c; any N stage; M0) Histological subtype—invasive carcinoma of non-specific type (IBC-NST; ICD-O: 8500/3) Triple-negative breast cancer (ER = 0; PR = 0; no HER2/neu amplification or overexpression) Sufficient tumor tissue for immunohistochemical analysis Anthracycline-based neoadjuvant chemotherapy (NACT)	Pregnancy at the time of sample collection Severe comorbid conditions (heart failure stage III–IV according to NYHA, sepsis, etc.) Synchronous or metachronous cancer History of prior malignant diseases Mutations in BRCA1; BRCA2; CHEK2 Incomplete NACT

**Table 2:** Nottingham grading score.

Criteria	Score		
	1	2	3
Gland formation	<10%	10–75%	>75%
Nuclear atypia	Weak	Intermediate	Prominent
Mitotic count *	≤12	12–24	>25
Total score	3–5	6–7	8–9
	Grade 1	Grade 2	Grade 3

Note: *—determined by hot spots in 10 high-power fields, with corrections for field of view area (data given for a diameter of 0.65 mm).

**Table 3:** Allred score for Estrogen and Progesterone Receptor evaluation.

Proportion Score	Positive Cells, %	Intensity/Score
0	0	No staining/0
1	<1	Weak/1
2	1–10	Intermediate/2
3	11–33	Strong/3
4	34–66	
5	≥67	

*Comment:* The final score = proportion score + intensity score. 0–2—negative result; 3–8—positive result.

**Table 4:** Assessment of HER2/neu expression level.

HER2/Status	Criteria
Negative (Score 0)	Absence of staining, or membrane staining that was incomplete and weak in ≤10% of tumor cells.
Negative (Score 1+)	Incomplete weal membrane staining in ≥10% of tumor cells.
Equivocal (Score 2+)	Weak or moderate complete membrane staining in ≥10% of tumor cells, or complete intense membrane staining in ≤10% of tumor cells.
Positive (Score 3+)	Complete intense membrane staining in >10% of tumor cells.

**Table 5:** Surrogate molecular-genetic subtypes of breast cancer.

Surrogate Molecular-Genetic Subtype	IHC Characteristics	Frequency of Occurrence
Luminal A	ER-positive; PR-positive; HER2-negative; Ki-67 ≤ 20% *	55%
Luminal B HER2-negative	ER-positive; HER2-negative and at least 1 of the following: Ki-67 ≥ 30%, PR < 20% (percentage of expressing cells)	15%
Luminal B HER2-positive	ER-positive; HER2-positive; Ki-67 any; PR any	
HER2-enriched	ER-negative; PR-negative; HER2-positive	15–20%
Triple negative	ER-negative; PR-negative; HER2-negative	10–15%

Note: *—Ki-67 cutoff values may be individual for each department. ER—estrogen receptor; PR—progesterone receptor. HER2—Human Epidermal Growth Factor Receptor 2.

**Table 6:** Clinicopathologic features of 161 patients with surgically resected TNBC. Correlation with NIS expression.

Variables	TNBC Samples (Total n = 161)				
	NIS Expression				
	NIS 0	NIS 1+	NIS 2+	NIS 3+	*p*-Value
Age (years) (n, %)					
<45	17 (10.56%)	6 (3.73%)	0	16 (9.94%)	0.073
≥45	32 (19.88%)	34 (21.12%)	24 (14.91%)	32 (19.88%)	
cT stage (n, %)					
1	1 (0.62%)	0	8 (4.97%)	0	0.507
2	40 (24.84%)	40 (24.84%)	8 (4.97%)	40 (24.84%)	
3	8 (4.97%)	0	0	0	
4	0	0	8 (4.97%)	8 (4.97%)	
cN stage (n, %)					
0	40 (24.84%)	16 (9.94%)	16 (9.94%)	40 (24.84%)	0.240
1	0	8 (4.97%)	8 (4.97%)	0	
2	0	8 (4.97%)	0	0	
3	9 (5.59%)	8 (4.97%)	0	8	
Lymphovascular invasion (n, %)					
Not identified	40 (24.84%)	16 (9.94%)	16 (9.94%)	40 (24.84%)	0.390
Present	9 (5.59%)	24 (14.9%)	8 (4.97%)	8 (4.97%)	
Residual cancer burden (n, %)					
0	33 (20.5%)	24 (14.9%)	16 (9.94%)	8 (4.97%)	<0.01
I	16 (9.94%)	16 (9.94%)	0	0	
II	0	0	8 (4.97%)	32 (19.87%)	
III	0	0	0	8 (4.97%)	
Chemotherapy regimen					
Anthracycline + taxane	16 (9.94%)	24 (14.9%)	16 (9.94%)	40 (24.84%)	<0.01 (NIS-RCB)
Anthracycline + taxane + carboplatin	33 (20.5%)	16 (9.94%)	8 (4.97%)	8 (4.97%)	<0.01 (NIS-RCB)

Abbreviations: TNBC—triple-negative breast cancer; RCB—residual cancer burden; NIS—sodium/iodide symporter.

## Data Availability

The original contributions presented in this study are included in the article material. Further inquiries can be directed to the corresponding author.
